# Hypothyroidism causing paralytic ileus and acute kidney injury - case report

**DOI:** 10.1186/1756-6614-4-7

**Published:** 2011-02-08

**Authors:** Chaturaka Rodrigo, Champika SSSK Gamakaranage, Dhanesha S Epa, Ariaranee Gnanathasan, Senaka Rajapakse

**Affiliations:** 1 University Medical Unit, National Hospital of Sri Lanka, Colombo, Sri Lanka; 2 Department of Clinical Medicine, Faculty of Medicine, University of Colombo, Sri Lanka

## Abstract

We present a patient with severe hypothyroidism complicated by paralytic ileus and acute kidney injury. A 65 year old male patient, diagnosed with hypothyroidism one year ago was transferred to our unit in a state of drowsiness and confusion. He was severely hypothyroid and had paralytic ileus and impaired renal function at the time of transfer. Hypokalaemia was present, and was likely to have contributed to the paralytic ileus and this together with dehydration was likely to have contributed to renal injury. Nonetheless, hypothyroidism is very likely to have been the principal precipitant of both these complications, and both paralytic ileus and acute kidney injury improved with thyroxine replacement. Unfortunately, the patient died unexpectedly eight days after admission to the unit.

Hypothyroidism may induce de novo acute kidney injury or it may exacerbate ongoing chronic kidney disease. This rare complication is assumed to be due to the hypodynamic circulatory state created by thyroid hormone deficiency. Paralytic ileus is an even rarer fatal manifestation of hypothyroidism and is thought to be due to an autonomic neuropathy affecting the intestines that is reversible with thyroxine replacement. To our knowledge, both these complications have not been observed in a single patient so far.

It is important that clinicians are aware of these rare manifestations of hypothyroidism as in most occasions, thyroxine deficiency may be missed, and treatment can reverse the complications.

## Background

Hypothyroidism presenting with acute kidney injury is rare, with only few cases reported so far [1,2]. Hypothyroidism may induce de novo acute kidney injury (AKI) or may exacerbate ongoing chronic kidney disease[3] or contribute to the occurrence of AKI in the presence of other renal insults. AKI is life threatening, and identification of possible contributory causes and early treatment is potentially life saving. Paralytic ileus is an even rarer fatal manifestation of hypothyroidism [4]. In this article we discuss a patient presenting with severe hypothyroidism and both these complications.

## Case presentation and discussion

### Case presentation

A 65 year old male patient, diagnosed with hypothyroidism one year ago, was transferred to our unit in a state of drowsiness and confusion. He did not have any other comorbidities and the cause for hypothyroidism was unknown. He had been on thyroxine replacement (150 μg daily) but had defaulted treatment for 3 months. In the months prior to admission, he had complained of constipation, lethargy, coarse skin and family members had noticed mental slowing, hoarse voice and changes in facial appearance. The patient was first admitted to a local hospital in a state of confusion and drowsiness with generalized body swelling, where oral thyroxine replacement was started. Coincidentally, he fell from his bed two days later and became unconscious. CT scan brain showed a small acute subdural haemorrhage over the right parietal lobe and he was transferred to the National Hospital, Colombo, Sri Lanka for neurosurgical opinion. While in the surgical unit, he developed progressive abdominal distension and absent bowel sounds. His urine output progressively declined and the serum creatinine was rising. He was then transferred to the University Medical Unit for medical management.

On admission to our unit, he had typical features of untreated hypothyroidism such as coarse myxoedematous skin, characteristic facial appearance, dry thin hair and oedema. He was not hypothermic but was dehydrated. His heart rate was around 60/min, blood pressure was 150/90 and there were no clinically detectable pericardial effusions or cardiac murmurs. His respiratory system was clinically normal. His abdomen was grossly distended with absent bowel sounds. There was no palpable organomegaly. He was confused and drowsy (Glasgow coma scale; E-4, M-6, V-4) and had slow-relaxing reflexes.

The records showed that at initial presentation to the local hospital, his TSH (Thyroid Stimulating Hormone) level was 40.5 mIU/μl (normal 0.4-4.0). Serum free thyroxine (FT_4 _) level was not available at that time. On admission to our unit (2 weeks after the initial presentation and while on thyroxine replacement), his TSH level had dropped to 29.9 mIU/μl, and the FT_4 _level was 0.61 ng/dl (0.89-1.76) - thus he was still severely hypothyroid. His serum creatinine had progressively risen from 122 μmol/l to 629 μmol/l with a concomitant drop in urine output (less than 30 ml per hour). He was normotensive throughout. There were no active urinary sediments and ultrasound scan abdomen showed normal sized kidneys with features of acute parenchymal disease together with bilateral fullness in the pelvicalyceal systems. The creatine phosphokinase level was not raised. He was not on any nephrotoxic drugs. At the onset of renal impairment, the patient had hyponatraemia (115-119 meq/l) and later on he was persistently hypokalaemic (2.5 - 3.4 meq/l). Abdominal radiographs showed grossly dilated bowel loops. He had a normal electrocardiogram but the echocardiogram showed grade II mitral regurgitation. There was no pericardial effusion. The patient's inflammatory markers were normal, and clinical features did not support peritonitis. Serum amylase was normal. We considered mechanical bowel obstruction, but found no clinical or investigation findings to support this; surgical opinion was sought, and a surgical acute abdomen was thought to be unlikely. Our diagnosis was paralytic ileus. We were unable to obtain a CT scan abdomen because of the dangers of contrast nephropathy aggravating existing AKI.

The patient was managed on the principles of thyroxine replacement and supportive therapy on the hypothesis that hypothyroidism and its sequalae were responsible for the clinical picture. Thyroxine was replaced (levothyroxine 200 μg/day as standard maintenance dose) via nasogastric tube and dehydration was corrected with carefully administered intravenous fluids with potassium replacement. Intravenous liothyronine was unavailable in our setting. With hydration and thyroxine replacement, the patient's renal functions improved (serum creatinine dropped to 278 μmol/l over 4 days) together with diuresis. Hyponatraemia also normalized without specific treatment. Dialysis was not required. Similarly, the bowel sounds returned and he opened bowels. Oral feeding was initiated with liquid feeds. The overall condition of the patient including the level of consciousness improved with thyroxine replacement. He was conscious, rational and was mobilized. However, on the 8^th ^day after transfer to our unit, he suddenly deteriorated and died. The cause for the unexpected death could not be established as the relatives refused a pathological post mortem. The cause of death could not be determined from the available investigation results at the time of death. The serum free T4 level was within normal range although the TSH level was still high (25.6 mIU/μl), and serum creatinine was 209 μmol/l immediately prior to the death. Serum electrolytes were normal for several days prior to death.

### Discussion

There are several case reports of both acute renal failure [1,5] and paralytic ileus[6,7] occurring in untreated hypothyroidism. However both these complications appearing in the same patient has not been reported.

The exact pathogenesis of acute kidney injury in hypothyroidism is still unclear and thought to be multi-factorial. However, the predominant mode of kidney injury is thought to be the reduced plasma flow and glomerular filtration rate due to the hypodynamic circulation[2]. The hypodynamic circulatory state results in a pre-renal insufficiency and this may be aggravated by other multi-systemic effects of hypothyroidism such as reduced cardiac output, low volume status, hyponatraemia with associated haemodynamic changes and increased peripheral resistance due to arterial wall stiffness[2]. However, this alone may not explain the extent of acute kidney injury. Primary glomerular and tubular dysfunction in hypothyroidism has also been observed with supportive histological evidence from biopsy specimens (thickening of glomerular and tubular basement membranes and inclusions in cell cytoplasm)[8]. These were reversible with thyroxine therapy. Rhabdomyolysis, another rare but known manifestation of hypothyroidism can also result in acute kidney injury but it is usually associated with another precipitating factor such as drugs or trauma[5]. Our patient however had no evidence of rhabdomyolysis. The available evidence suggests that renal impairment may start as quickly as two weeks in to the hypothyroid state and it recovers fully with thyroxine replacement[9]. The long term impact (if any) of hypothyroidism on renal function is unknown[5].

Paralytic ileus in hypothyroidism is assumed to be due to an autonomic neuropathy affecting the extrinsic nerves of the colon[10]. There are only a few cases of this complication reported in literature with the first one being reported by Bastenie in 1946[7]. Bastenie hypothesized that myxoedematous material deposition in the muscle fibers of intestines interfered with their integration with autonomic ganglia. Later in 1969 and 1977, two case reports of death due to paralytic ileus with hypothyroidism were published[11,12]. In the report by Wells et al[11], the patient died after 20 days since presentation and the post mortem at that time gave an insight in to the possible pathogenesis in this unusual complication. Histological sections revealed gross abnormalities in extrinsic nerves entering the intestines while some less prominent changes were also observed in intrinsic plexuses. The authors suggest that the mechanism may be an autonomic neuropathy similar to the peripheral neuropathy frequently observed in hypothyroidism.

Surgical intervention for hypothyroidism induced paralytic ileus is not recommended as the neuropathy is reversible with thyroxine replacement. However, atonia may take time to reverse and the patients can succumb to complications of ileus[4].

In addition to paralytic ileus, patients may also present with urinary retention and this observation is also taken as supportive evidence for the autonomic neuropathy in hypothyroidism [4]. The fullness of the pelvicalyceal systems observed on ultrasound scan of our patient was possibly a result of this. We were unable to find any published data as to how the presence of paralytic ileus would have affected the absorption of thyroxine given via NG tube; the non-availability of intravenous liothyronine was a definite, albeit unavoidable, shortcoming in our management.

In the timeline of events, it is noted that the paralytic ileus preceded the acute kidney injury. The fluid sequestration in the bowels would have led to severe dehydration, hyponatraemia and hypokalaemia. Given the patient's background and sequence of events, it is likely that hypothyroidism was the primary cause of paralytic ileus though subsequent hypokalaemia undoubtedly contributed to making it worse. It is unlikely that hypokalaemia was the primary cause of paralytic ileus since hypokalaemia developed later on. The acute kidney injury in this patient is unlikely to be due to rhabdomyolysis as there was no supportive clinical or laboratory evidence. Instead, it is very likely that reduced renal plasma flow caused by hypothyroidism and the fluid sequestration within the intestines due to paralytic ileus in combination resulted in AKI. The paralytic ileus responded to potassium and thyroxine replacement and the concomitant vigorous fluid management would have improved the renal plasma flow. Both these therapeutic measures would have contributed to the rapid recovery of renal function. The relatively rapid recovery of renal function supports hypothyroidism related AKI rather than acute tubular necrosis due to dehydration.

In this patient, the root cause for paralytic ileus and acute kidney injury was the lack of thyroxine. The patient was managed on thyroxine replacement and supportive therapy leading to clinical improvement without surgery or invasive procedures, thus with hindsight our diagnosis of paralytic ileus was correct. The cause of sudden death in this patient may either be related to hypothyroidism and its complications or caused by an independent factor. Though kidney injury reverses fast with thyroxine replacement, the smooth muscle atonia is believed to take a longer time for recovery, and complications of ileus might have contributed to death [11,12]. Although hyponatraemia and hypokalaemia were present initially, both serum sodium and potassium levels were normal for several days prior to his deterioration, and are hence unlikely to have been responsible for his death. On the other hand, an arrhythmia or pulmonary embolism (the patient was bedridden for over 3 weeks) or possibly an acute rebleed into the existing subdural haemorrhage (with raised intracranial pressure and coning) are possible causes for his death.

## Conclusions

It is important that clinicians are aware of the rare manifestations of hypothyroidism such as acute kidney injury and paralytic ileus. The easily reversible thyroxine deficiency may be missed when patients present with such complications unless there is an obvious past history.

## Consent

Written informed consent was obtained from the patient's relatives for publication of this case report. A copy of the written consent is available for review by the Editor-in-Chief of this journal.

## List of abbreviations

Abbreviations are explained where they are first used in text

## Competing interests

The authors declare that they have no competing interests.

## Authors' contributions

CR, CG and DSE collected information on the patient. CR wrote the first draft. SR and CR did the literature searches and wrote the final manuscript and made appropriate revisions. All authors read through and approved the final manuscript.

## Authors' information

CR and CG (MBBS) are registrars in Internal Medicine attached to the University Medical Unit of the National Hospital of Sri Lanka. DSE (MBBS) is the House Officer of the same unit. AG (MBBS, MD, MPhil, FRCP) is Consultant Physician and Senior Lecturer attached to the Department of Clinical Medicine, Faculty of Medicine, University of Colombo, Sri Lanka. SR (MBBS, MD, FRCP, FCCP, FACP) is Consultant Physician and Professor in the same department.
